# A diagnostic challenge presented in a 37-year-old man with severe weight loss and multiple liver masses 

**Published:** 2020

**Authors:** Saeed Abdi, Amin Momeni Moghadam, Mitra Rafiezadeh, Forough Mangeli, Ayub Ghafurian

**Affiliations:** 1 *Gastroenterology and Liver Diseases Research Center, Research Institute for Gastroenterology and Liver Diseases, Shahid Beheshti University of Medical Sciences, Tehran, Iran.*; 2 *Taleghani Hospital, Shahid Beheshti University of Medical Sciences, Tehran, Iran.*; 3 *Basic and Molecular Epidemiology of Gastrointestinal Disorders Research Center, Research Institute for Gastroenterology and Liver Diseases, Shahid Beheshti University of Medical Sciences, Tehran, Iran*

## Question

**Figure 1:**
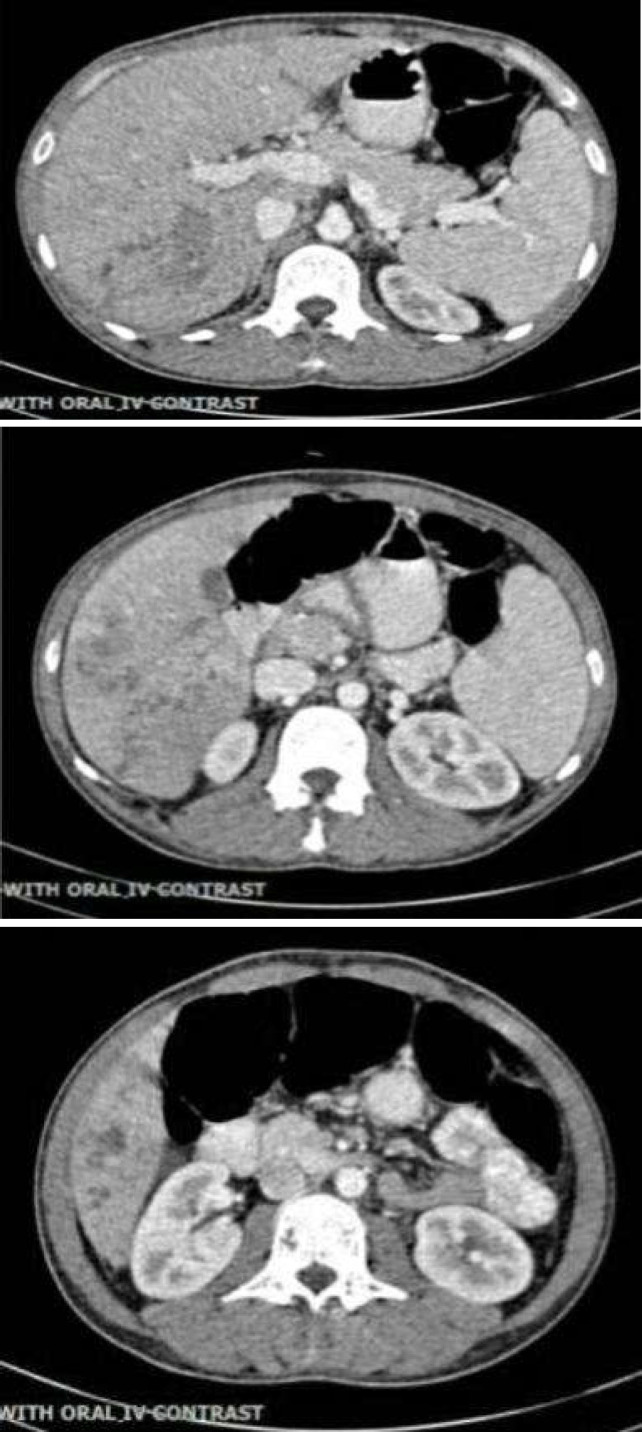
Abdominopelvic CT scan demonstrates disseminated intrahepatic duct dilation and periductal enhancement of the liver parenchyma predominantly in right liver lobe

**Figure 2:**
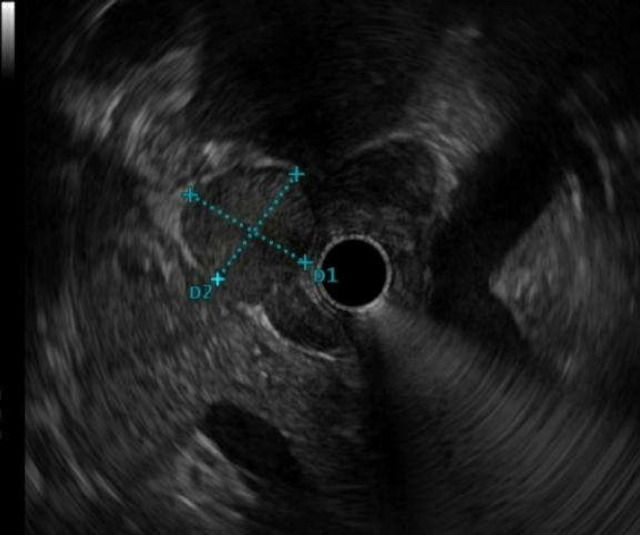
A hypoechoic lymph node in the hilum

 A 37-year-old man, Iranian resident in Zanjan, presented to the gastroenterology clinic of Taleghani Hospital; a teaching referral hospital in Tehran, Iran, with chief complaint of a significant weight loss about 20 kg in recent 4 months. He denied any concomitant systemic signs and symptoms except for a generalized malaise. He did not mention any change in bowel habit and eating pattern but a slight decrease in appetite without nausea and vomiting. He denied smoking and use of any illicit drug or alcohol. His past medical history was negative. He was admitted for further work regarding his unexplained weight loss. On admission, he was well with stable vital signs. His blood pressure was 110/70 mmHg, body temperature: 36.5^ o ^C, heart rate: 84 bites per minute, and respiratory rate: 13 per minute all within normal limits. His physical exam was only notable for a mild epigastric and right upper quadrant (RUQ) tenderness. His laboratory tests were normal except for a normochromic normocytic anemia with a hemoglobin concentration of 12 mg/dl. Serum iron level was 37 mcg/dl with a total iron binding capacity (TIBC) level of 275 mcg/dl and a ferritin level of 10 mcg/l revealing an iron deficient erythropoiesis. Further laboratory tests evaluating tumor markers, thyroid function tests, liver function tests, serum bilirubin level, urine analysis and stool examination were all normal. We decided to proceed with an upper gastrointestinal endoscopy and colonoscopy in the next step of the management of the patient with significant weight loss. Total colonoscopy was unremarkable and esophagogastroduodenoscopy demonstrated a sessile polyp in antrum pathologically consistent with hyperplastic polyp. His spiral chest CT scan was normal but an abdominopelvic CT scan revealed dilation of intrahepatic ducts disseminated all over the liver prominently in the periphery of the right hepatic lobe. Periductal parenchyma was enhanced in some parts indicative of infective or neoplastic processes. Scattered celiac and mesentric lymph nodes with maximum diameter of 6 mm were also detected (figure 1). To have a better visualization of extrahepatic ducts besides intrahepatic ducts, an endoscopic ultrasound (EUS) was performed. As reflected in figure-2, findings of EUS were unremarkable except for 3 hypoechoic and round hilar lymph nodes with the largest diameter of 21mm, most probably indicative of reactive lymph nodes. This patient’s signs and symptoms continued to remain stable, so he was discharged and was advised to follow other diagnostic modalities in the outpatient clinic. Some months later, he referred to the gastroenterology clinic with similar complaints of inability to weight gain and anorexia. Given the previous history of intrahepatic duct dilation, an MRCP was demanded which revealed heterogeneity of the liver parenchyma due to multiple masses or microabscesses in the liver. Laboratory results were negative for infectious, helminthic and parasitic disorders for this kind of liver involvement, and imaging reports were unable to rule out a neoplastic process and give a definite diagnosis. This made us proceed with a liver biopsy. 

**What is your diagnosis?**

**What is the next step?**

## Discussion

**Figure 3:**
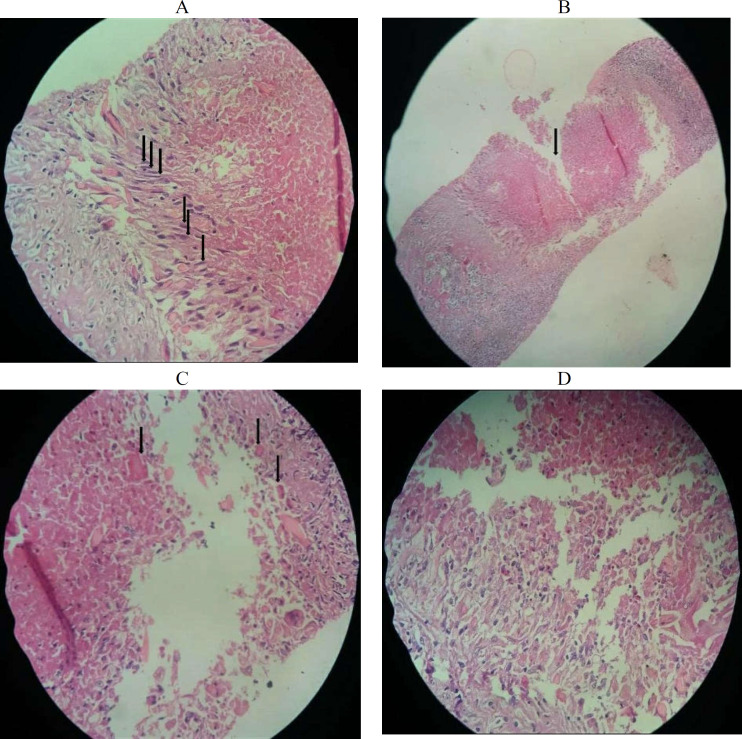
(A) Palisading macrophages perpendicular to the edge of the central necrosis, (B) Central necrosis, (C) Charcot-leyden crystals, and (D) Numerous eosinophils

Liver specimen revealed the liver parenchyma with focal lobular necrosis along with peripheral palisading spindle shaped epithelioid cells surrounded by moderate inflammatory cells mainly plasma cells, lymphocytes and eosinophils admixed with scattered foreign body type giant cells mainly compatible with Fasciola hepatica (figure 3). Prior to labelling this patient as having Fasciola hepatica infection, serial stool exams for the ova of Fasciola hepatica were demanded which confirmed the diagnosis.

Fascioliasis is caused by a trematode liver fluke which is called Fasciola hepatica (1). This infection has been detected all over the world. Iran is one the known countries with a significant report of infected cases (2). This trematode initially infects livestock. Their eggs will pass into the feces and will find their way into the fresh water where they will start their larval stage in their intermediate host which is a snail. Multiplication takes place within snails, then encysted metacercariae will be attached to vegetables and will be ingested by definitive host (herbivores) or accidental host (humans). Their journey through the human gastrointestinal tract will continue as they leave their cysts and they penetrate into the intestine, peritoneal cavity and finally liver capsule where they can experience two stages in the liver parenchyma and biliary tree. This journey takes some months to be completed, so the classic presentation of helminthiasis like eosinophilia and ova detection in the stool exam should not be expected at any time after the entrance of fluke into the gastrointestinal tract. The presented case is a young man admitted with severe weight loss and generalized malaise without signs, symptoms and laboratory tests indicative of a helminthic infection making the diagnosis a challenge to physicians. Having entered into the liver, metacercariae experience two stages. Initially, the organism migrates into parts of the hepatic parenchyma. The migration starts 1-3 months after ingestion of metacercaria and is followed by systemic and laboratory signs and symptoms of a parasitic infection like fever, allergic reactions, RUQ pain and eosinophilia. The presented case was admitted with weight loss and generalized malaise in this stage. This stage would also be complicated by acute hepatitis and subcapsular hemorrhage (3-5) The second stage starts when the organism is found to be in the biliary tree and is manifested by RUQ pain with or without cholangitis and cholestasis (6). Stool exam suffers from poor sensitivity for Fasciola hepatica eggs particularly in the first months of infection. Therefore, we have to bear in mind that the diagnosis of fasciola hepatica should not be ruled out with one negative stool exam for ova of parasites as could be seen in our patient. The method of choice in this stage when eggs are not detected in the stool exam is ELISA which detects antibody against antigenic products of Fasciola hepatica (7). Imaging modalities like ultrasound and CT scan are typical in the second stage of the liver involvement when the organism has encroached upon the biliary tract. Common bile duct (CBD) irregularity, CBD dilation, periductal fibrosis, small and hypodense lesions resembling micro abscesses in a branched pattern most commonly in subcapsular region are detected at this stage as reflected in the presented case (8,9,10).Magnetic resonance cholangiopancreatography (MRCP) usually shows the same findings (11,12). Liver biopsy demonstrates linear destruction of the parenchyma with necrosis, Charcot-leyden crystals, granuloma, fibrosis and cellular infiltration enriched by polymorphonuclears (PMN) and eosinophils. The association of Fasciola hepatica with liver fibrosis, cirrhosis and cancer is on debate. In the systematic review of Claudia Machicado et al. in 2016 (13), the association between Fasciola hepatica with liver fibrosis and cirrhosis has been highlighted but its relation to liver cancer has been controversial requiring more studies. The mechanism for liver fibrosis has been attributed to the activation of hepatic stellate cells by the cathepsins of the parasite in animal models (14). But, it cannot be definitely extrapolated to humans. This fact is well worth the follow-up of patients with fascioliasis to detect whether they progress to the liver fibrosis or not. It is essential to follow up patients without other risk factors of liver fibrosis like alcohol consumption and viral hepatitis as could be seen in our patient. Regarding the association of fascioliasis and liver cancer, there are some studies reflecting the overexpression of TGF-β and increasing mutations in mice. Nevertheless, there is still no documented human study to show the mentioned association except for a report in Peru (15). This study and similar studies in Peru revealed an association between unusual K-RAS mutations and cholangiocarcinoma attributed to the Fasciola hepatica which was not proved in other studies (16,17). The focus of this study is to prevent a delay in the diagnosis of fascioliasis. To proceed with an earlier diagnosis, it is logical to check serology for Fasciola hepatica if elevated liver enzyme and eosinophilia are accompanied by multiple hypodense lesions in the liver. 

Notably, the shortcoming seen in previous studies is the follow-up of cases treated or left untreated with Fasciola hepatica. Our patient was started on triclabendazole. In a 1-year follow-up of the patient, no fibrosis, cirrhosis or malignancy was detected. It seemed that the liver changes in our patient was reversible and it was completely cured. It implies by no means that patients with asymptomatic chronic infection of fascioliasis have no inflammation (18). A serum lipid peroxidation increase and an antioxidant enzyme decrease in these patients have been detected indicating the fact that a mild inflammation still exists in these patients (19). Importantly, an effective antiparasitic therapy alone does not protect patients from the progression of the disease based on previous studies (20) Therefore, more studies are required for long-term effects of Fasciola-hepatica on liver with and without treatment.
